# Evaluation of the effects of Plasma-Lyte 148 or 0.9% saline on acid-base balance in patients undergoing neurosurgery: a randomized clinical trial

**DOI:** 10.31744/einstein_journal/2026AO1574

**Published:** 2026-03-30

**Authors:** Murilo Moreira Thom, Guilherme Antonio Moreira de Barros, Lucas Guimarães Ferreira Fonseca, Rodrigo Leal Alves, Murillo Gonçalves Santos, Vitória Mariah Giriboni, Paulo do Nascimento, Norma Sueli Pinheiro Módolo

**Affiliations:** 1 Department of Anesthesiology Faculdade de Medicina Universidade Estadual Paulista Botucatu SP Brazil Department of Anesthesiology, Faculdade de Medicina, Universidade Estadual Paulista, Botucatu, SP, Brazil.

**Keywords:** Neurosurgery, Perioperative fluid management, Balanced crystalloids, Plasma-Lyte 148, Hyperchloremia, Acid-base homeostasis

## Abstract

**Objective:**

The choice of crystalloid solution considerably affects perioperative outcomes in neurosurgery. This study aimed to evaluate the effects of Plasma-Lyte 148 and 0.9% saline on acid-base and hydroelectrolyte balance in adults undergoing elective neurosurgical procedures.

**Methods:**

A double-blind randomized controlled trial was conducted at Botucatu Medical School from November 2019 to May 2020. Patients received either Plasma-Lyte 148 or 0.9% saline at a maintenance rate of 2mL/kg/h, with additional bolus guided by mean arterial pressure and pulse pressure variation. The primary endpoint was arterial pH at the end of surgery.

**Results:**

Sixty-eight patients (33 in the Plasma-Lyte 148 group and 35 in the saline group) completed the study. The total median volumes administered (1st-3rd quartiles) were 2,227 (1,416-3,000) and 3,000 (2,000-4,000) mL in the Plasma-Lyte 148 and saline groups, respectively (p=0.107). At procedure completion, Plasma-Lyte 148 demonstrated superior acid-base homeostasis with significantly higher pH (7.39±0.04 *versus* 7.35±0.05, p<0.001), bicarbonate (22.5±1.8 *versus* 20.6±2.2mmol/L, p<0.001), and base excess (−1.6±2.3 *versus* −3.9±2.6, p<0.001), while preventing hyperchloremia (109.1±6.6 *versus* 113.9±4.5 mmol/L, p=0.001).

**Conclusion:**

Plasma-Lyte 148 provides superior acid-base homeostasis compared to 0.9% saline in patients undergoing neurosurgery, with significantly better pH, bicarbonate, and base excess profiles while avoiding hyperchloremia. These findings support the preferential use of balanced crystalloids in neurosurgical practice, aligning with current evidence favoring Plasma-Lyte 148 for optimal perioperative fluid management. ReBEC platform registration number: RBR-2592-hd.

## HIGHLIGHTS

This randomized controlled trial in elective neurosurgery adds important evidence to the growing body of literature comparing Plasma-Lyte 148 with 0.9% saline in specialized surgical populations.

Plasma-Lyte 148 demonstrated superior acid-base homeostasis with significantly higher bicarbonate levels and base excess at surgical completion, whereas 0.9% saline induced hyperchloremia in the saline group.

The biochemical advantages of Plasma-Lyte 148 align with current evidence supporting balanced crystalloids as the preferred choice in perioperative fluid management.

These findings support the emerging consensus favoring balanced crystalloids over normal saline, particularly in populations where acid-base homeostasis is critical.

Our findings contribute to the paradigm shift toward Plasma-Lyte 148 as the preferred crystalloid solution in neurosurgical practice, consistent with recent international guidelines. while preventing hyperchloremia (p≤0.001).

## In Brief

This double-blind, randomized trial compared Plasma-Lyte 148 with 0.9% saline in 68 patients undergoing neurosurgery. Plasma-Lyte 148 demonstrated superior acid-base homeostasis with significantly higher pH (7.39 versus 7.35), bicarbonate (22.5 versus 20.6 mmol/L), and base excess (−1.6 versus −3.9),

## Graphical abstract

**Figure:**
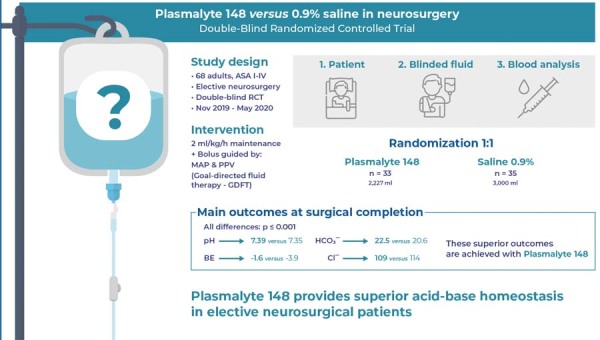

## INTRODUCTION

Intravenous fluid therapy is one of the most fundamental interventions in modern medicine and is administered daily to millions of patients worldwide for volume replacement, hemodynamic optimization, and medication delivery.^( 1 )^

In neurosurgery, where the maintenance of cerebral homeostasis is critical, appropriate crystalloid selection is particularly important, influencing not only acid-base and electrolyte balance but also intracranial pressure, cerebral perfusion, and neurological outcomes.^( 2 - 4 )^ Despite this universal relevance, the choice between balanced crystalloids and 0.9% saline remains one of the most debated issues in contemporary anesthesiology and perioperative medicine, with profound implications for patient outcomes across diverse clinical settings.^( 5 - 7 )^

Over the past two decades, scientific evidence regarding crystalloids has evolved substantially. The pioneering study by Yunos et al. first demonstrated that a chloride-restrictive strategy significantly reduced the incidence of acute kidney injury by 40% (8.4% *versus* 14%, p<0.001) in 1,533 critically ill patients.^( 8 )^ This finding was subsequently validated on a much larger scale by the Isotonic Solutions and Major Adverse Renal Events Trial (SMART) and Saline against Lactated Ringer’s or Plasma-Lyte in the Emergency Department (SALT-ED) studies (2018), which demonstrated consistent renal protection with balanced crystalloids in more than 29,000 patients. The SMART study, involving 15,802 critically ill patients, showed a significant reduction in major adverse kidney events (14.3% *versus* 15.4%, p=0.04), whereas the SALT-ED study, involving 13,347 non-critically ill patients, confirmed similar renal protection (4.7% *versus* 5.6%, p=0.01).^( 9 , 10 )^

However, more recent high-impact studies have refined this body of evidence and challenged the long-held assumption that balanced crystalloids are universally superior to 0.9% saline. The Balanced Solution versus Saline in Intensive Care Study (BaSICS; 2021), involving 10,520 critically ill patients, found no significant difference in mortality (26.4% *versus* 27.2%, p=0.47), raising specific concerns about balanced crystalloids in traumatic brain injury.^( 11 )^ This concern was amplified by recent meta-analyses, including the study by Diz et al., which demonstrated increased 90-day mortality with balanced crystalloids in patients with traumatic brain injury (odds ratio [OR] 1.31, 95% confidence interval [95%CI] 1.03-1.65, p=0.03).^( 12 )^ The Plasma-Lyte 148 *versus* Saline (PLUS) study (2022), one of the most recent and rigorous double-blind clinical trials involving 5,037 critically ill patients, confirmed this equipoise by demonstrating no significant difference in mortality (21.8% *versus* 22.0%, p=0.90) or renal function between Plasma-Lyte 148 and saline.^( 13 )^

Despite the extensive literature in general critical populations, a substantial knowledge gap persists in the context of elective neurosurgery.^( 14 , 15 )^ Unlike traumatic brain injury, where primary brain injury, edema, intracranial hypertension, and hemodynamic instability create a complex pathophysiological context, elective neurosurgery occurs under controlled conditions in stable patients with an intact blood-brain barrier.^( 16 , 17 )^ This distinction is crucial, as the proposed mechanisms explaining the adverse effects of balanced crystalloids in traumatic brain injury, including cerebral edema, intracranial pressure alterations, and blood-brain barrier dysfunction, may not apply to the elective context.^( 18 , 19 )^

The few specific studies in elective neurosurgery have consistently demonstrated the metabolic advantages of balanced crystalloids. In a study of 80 patients undergoing elective craniotomy, Shrivastava et al. found that Plasma-Lyte was associated with a significantly higher pH (7.42 *versus* 7.38, p<0.001) and a lower incidence of metabolic acidosis.^( 20 )^ Similarly, Dey et al. found identical results in 44 patients, with superior arterial pH (7.41 *versus* 7.36, p<0.001) and a better electrolyte profile with Plasma-Lyte.^( 21 )^ Kang et al., in a study involving 586 patients undergoing aneurysm clipping, demonstrated not only metabolic advantages but also superior clinical outcomes, including earlier extubation (122 *versus* 250 min, p=0.016) and shorter intensive care unit (ICU) stay (1.12 *versus* 1.37 days, p=0.001) with balanced crystalloids.^( 22 )^

The physiological basis for these metabolic advantages is well-established. A 0.9% saline solution with a chloride concentration of 154mEq/L (substantially higher than plasma’s 103mEq/L) induces hyperchloremic acidosis through reduction of the strong ion difference, as described by Stewart’s approach.^( 23 , 24 )^ In contrast, balanced crystalloids, such as Plasma-Lyte 148, with a chloride concentration of 98 mEq/L and presence of metabolic buffers (acetate and gluconate), maintain acid-base balance closer to physiological conditions.^( 25 )^ Recent experimental studies, including work by Bessa et al. involving an animal model of ischemic stroke, have demonstrated that different sodium concentrations in fluids considerably affect brain, lung, and kidney injury, suggesting that an optimized electrolyte composition may have neuroprotective implications.^( 26 )^

Considering this gap in the literature and the growing importance of individualized fluid therapy based on clinical context, we conducted this randomized, double-blind controlled trial to directly compare the effects of Plasma-Lyte 148 and 0.9% saline on acid-base balance, electrolyte profile, and perioperative outcomes in patients undergoing elective neurosurgery. Our primary hypothesis was that Plasma-Lyte 148, owing to its more physiological composition, would offer superior acid-base homeostasis compared to 0.9% saline, as measured by arterial pH at the end of surgery. By providing meaningful metabolic advantages without compromising safety, Plasma-Lyte 148 could help refine current evidence in the elective neurosurgical setting. In this context, balanced crystalloids may deliver benefits under controlled conditions while maintaining comparable safety, thereby informing clinical practice and guiding future guidelines in this specialized field.

## OBJECTIVE

To evaluate the effects of Plasma-Lyte 148 and 0.9% saline on acid-base balance, electrolyte profile, and perioperative outcomes in patients undergoing elective neurosurgery, with arterial pH at the end of surgery as the primary endpoint.

## METHODS

### Ethics statements

This study was approved by the Research Ethics Committee (CAAE: 96280018.9.0000.5411; # 2.879.197; September 6, 2018) of the *Faculdade de Medicina de Botucatu, Universidade Estadual Paulista* . The trial was prospectively registered in the Brazilian Clinical Trials Registry on October 20, 2019, prior to patient enrollment, ensuring transparency and methodological rigor in accordance with international standards for clinical research reporting.^( 27 , 28 )^ The study was conducted in strict adherence to the principles of the Declaration of Helsinki and followed the Consolidated Standards of Reporting Trials (CONSORT 2010) guidelines for randomized controlled trials.^( 29 , 30 )^ Written informed consent was obtained from all participants or their legally authorized representatives before enrollment in the study.

### Study design and population

This double-blind, randomized controlled clinical trial was conducted at the Hospital de Botucatu Medical School between November 2019 and May 2020. The study design employed rigorous methodological standards to minimize bias and ensure the validity of findings in this specialized neurosurgical population.^( 31 , 32 )^

Eligible participants were adults aged 18 years or older undergoing elective intracranial neurosurgical procedures, including tumor resection or cerebral aneurysm clipping, and classified as American Society of Anesthesiologists (ASA) physical status I-IV. This broad ASA classification range was deliberately chosen to enhance the external validity of our findings while maintaining safety standards appropriate for major neurosurgical interventions.^( 33 , 34 )^

The following criteria were defined to eliminate confounding variables that could interfere with the assessment of acid-base and electrolyte homeostasis. We did not include patients with cardiac arrhythmia (which could affect hemodynamic stability and fluid management), chronic kidney disease (which could alter electrolyte handling and acid-base regulation), previously diagnosed acid-base or electrolyte abnormalities (to ensure baseline comparability), pregnancy (due to physiological changes in fluid distribution and acid-base status), or current diuretic use (which could confound fluid balance assessment).^( 35 , 36 )^ Additionally, we excluded patients who dropped out after randomization, were lost to follow-up, or required transfusion of more than four packed red blood cells (pRBC) in 1 h or 10 pRBC in 24 h, as this would substantially alter the hemodynamic and metabolic milieu beyond the scope of crystalloid comparison.^( 37 - 39 )^

Patients were randomized in a 1:1 ratio into two groups (0.9% saline and Plasma-Lyte 148) according to the fluid replacement protocol, with codes generated by computer software (random.org) to ensure equal distribution between treatment groups. Allocation concealment was ensured by placing group assignments in sealed, opaque envelopes kept confidential by an individual not involved in the study.^( 40 , 41 )^

Before each procedure, a member of the hospital pharmacy opened the envelope corresponding to the study group and prepared the assigned solution. To maintain blinding, the fluid containers were covered with opaque black bags labeled with a kit number and bar code, ensuring that patients received only the randomized fluid, and that the attending anesthesiologist remained blinded.^( 42 , 43 )^

Physicians responsible for evaluating the laboratory test results and possible complications were blinded to the patient grouping until the end of the study.

### Surgical procedures

We conducted a comprehensive preoperative evaluation for all participants, including a detailed anesthetic assessment and informed consent documentation. This standardized approach ensured optimal patient preparation and baseline risk stratification.^( 44 , 45 )^

Once in the operating theater, patients were monitored with continuous cardioscopy, pulse oximetry, and noninvasive blood pressure measurement. After induction of anesthesia, an arterial catheter was placed for invasive blood pressure monitoring and measurement of pulse pressure variation (PPV). A central venous access was established for administering vasoactive drugs when clinically indicated.^( 46 , 47 )^

Anesthetic depth was monitored using bispectral index (BIS) technology to ensure an optimal hypnotic state throughout the procedure, reflecting current best practices in neuroanesthesia monitoring.^( 17 , 48 )^ The anesthetic protocol followed evidence-based guidelines and incorporated contemporary approaches to neurosurgical anesthesia management.^( 49 )^

Mechanical ventilation was optimized using lung protective strategies with tidal volumes of 8mL/kg of predicted body weight, calculated using established formulas (height in cm: 100 for men and 105 for women), consistent with current perioperative ventilation recommendations.^( 50 , 51 )^ Respiratory rate was adjusted to maintain arterial carbon dioxide tension between 30 and 35 mmHg, targeting mild hypocapnia, as appropriate for intracranial procedures.^( 30 , 52 )^ Positive end-expiratory pressure was individualized based on lung compliance assessment, typically ranging from 3 to 8cmH_2_O to optimize oxygenation while minimizing cardiovascular compromise.^( 53 , 54 )^

**Figure 1:**
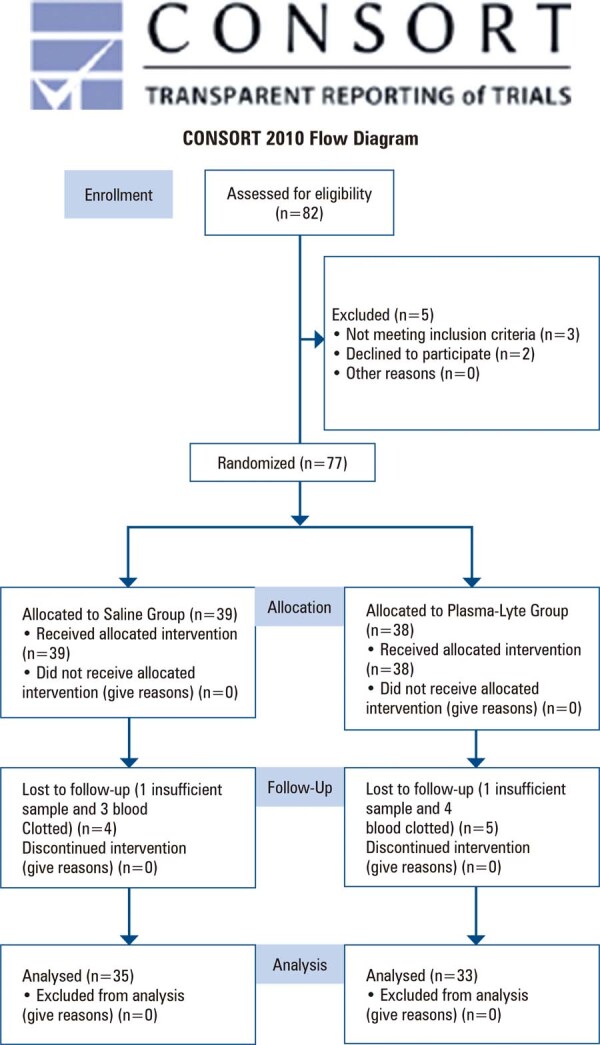
Consolidated Standards of Reporting Trials diagram of patient recruitment

Intraoperative fluid was administered according to a goal-directed therapy (Figure 1S, Supplementary Material ), incorporating contemporary evidence on hemodynamic optimization in patients undergoing neurosurgery.^( 55 , 56 )^ The protocol utilized mean arterial pressure and PPV as primary hemodynamic targets, reflecting current understanding of fluid responsiveness assessment in mechanically ventilated patients.^( 57 , 58 )^

The randomized crystalloid was infused continuously via an infusion pump at a rate of 2mL/kg/h^( 59 )^ of ideal body weight^( 60 )^ during the entire procedure until the end of dressing, when it was interrupted. The anesthesiologist’s assistant administered only the randomized crystalloid fluid. Adjustments to the anesthetic plan were made according to variations in PAM and BIS, and included a 200 mL fluid bolus over 10 min (pump 2), pRBC transfusion, vasopressor bolus, or continuous norepinephrine infusion (Figure 1S, Supplementary Material ). This approach was guided by the standardized protocol to minimize inter-provider variability.

Notably, the drugs and medications administered were diluted exclusively with the randomized solution. Thus, the patients differed only in the type of crystalloid solution administered.

### Data collection and study variables

The following variables were assessed: age, sex, weight, height, ASA classification, surgical indication, duration of surgery and anesthesia, volume of crystalloid solution, diuresis, fluid balance, plasma electrolytes and lactate levels, glycemia, arterial pH, HCO3^-^ and base excess, transfusion of pRBC, doses of ephedrine and metaraminol, use of norepinephrine, early extubation in the operating room, length of stay in the ICU and hospital, new neurological deficits, and mortality.

Laboratory reference values were established according to current clinical practice guidelines (Table 1S, Supplementary Material ,), ensuring standardized interpretation of biochemical parameters.^( 61 , 62 )^

### Outcomes

The primary endpoint was arterial blood pH at the end of surgery, representing the most direct assessment of acid-base homeostasis following crystalloid administration.^( 63 , 64 )^ This endpoint was selected based on its clinical relevance and sensitivity for detecting the metabolic effects of different crystalloid solutions in the perioperative setting. Secondary endpoints were the dosage of HCO3^-^, base excess, and plasma electrolytes; extubation in the operating room; new neurological deficits; and mortality. Moreover, secondary outcomes such as hospital or ICU length of stay, new neurological deficit, and death were assessed during the first 7 postoperative days. These comprehensive secondary outcomes were designed to capture both biochemical and clinically relevant effects of crystalloid selection in patients undergoing neurosurgery.

### Statistical analysis

#### Sample size calculation

The sample size was calculated based on the meta-analysis by Huang et al.,^( 65 )^ which compared 0.9% saline with balanced crystalloid solutions in adults undergoing non-renal surgeries. Considering a mean standard deviation of 0.05 for postoperative pH and a clinically relevant difference of 0.035 points in the pH between groups at the end of surgery,^( 66 )^ a minimum of 33 patients in each group was necessary to test the main hypothesis, providing 80% power of detection and a type 1 error of 5%.

#### Statistical description

Qualitative variables are expressed as absolute and relative frequencies. Quantitative variables are expressed as mean and standard deviation (normal distribution) or median and quartiles (non-normal distribution). The normality of the distribution of quantitative variables was evaluated from the histogram and Q-Q plot analysis using the Shapiro-Wilk test.

The χ^2^ and Fisher’s exact tests were used to compare qualitative variables between groups, whereas the Student’s t-test was used to compare the means of continuous variables with normal distribution. The Mann-Whitney U test and Hodges-Lehmann median difference estimate were used to analyze continuous or discrete variables with non-normal distributions.

All differences between outcome variables are presented with their respective 95% CI. Statistical significance was set at p<0.05. All analyses were performed using IBM SPSS Statistics version 22.0 (IBM Corp., Armonk, NY, USA).

## Validation of the study

Study reporting adhered to the CONSORT 2010 guidelines for randomized controlled trials.^( 67 )^ Methodological quality was assessed using contemporary risk of bias evaluation tools, ensuring transparency and reproducibility of findings.^( 27 , 49 , 68 )^

## RESULTS

A total of 68 patients completed the study and were included in the per-protocol analysis: 35 patients in the 0.9% saline group and 33 in the Plasmalyte 148 group ( Figure 1 ). The results presented represent the outcomes of patients who reached the end of the study according to their initial allocation (per protocol).

Baseline demographic and clinical characteristics were similar between groups, including age, sex distribution, weight, height, ASA classification, and surgical indication. Baseline acid-base and electrolyte values were also similar between groups ( Table 1 ).

**Table 1:** Demographic data and baseline conditions

Variable	0.9% Saline Group (n=35)	Plasma-Lyte Group (n=33)
Demographic		
Age (years)^*^	50.6±15.6	48.3±14.1
Gender (Male/Female)^†^ - %	15/20 (42.9/57.1)	13/20 (39.4/60.6)
Weight (kg)^*^	73.5±14.5	74.8±13.6
Height (cm)^*^	1.67±0.10	1.65±0.12
ASA (I/II/III/IV)^†^	0/17/16/2	2/11/18/2
Surgical indication (Tumor/Aneurysm)^†^ - %	24/11 (68.6/31.4)	24/9 (72.7/27.3)
Changes in ECG^*^ - %	2 (5.7)	3 (9.1)
Changes in X-ray^*^ - %	2 (5.7)	1 (3.0)
Baseline values		
pH^*^	7.37±0.05	7.40±0.04
HCO_3_^-^ (mmol/L)^#^	22.9±2.5	23.6±2.7
Base Excess^*^	-1.2±2.7	-0.4±2.6
Sodium (mmol/L)^#^	139.2±2.8	138.9±3.2
Potassium (mmol/L)^#^	3.9±0.3	3.9±0.3
Calcium (mmol/L)^#^	1.18±0.04	1.16±0.04
Chlorine (mmol/L)^#^	109.1±3.1	108.8±2.7
Lactate (mmol/L)^§^	1.0 (0.8/1.4)	1.0 (0.7/1.4)
Blood Glucose (mg/dL)^§^	107 (100.0/132.0)	102 (92.0/114.0)

^*^ Values are expressed as mean±standard deviation and were analyzed using the Student’s t-test; ^†^ Values are expressed as absolute and relative frequencies and were analyzed using the Chi-square and Fisher exact tests; ^§^ Values are expressed as mean±standard deviation; ^¶^ Values are expressed as median with quartiles.

ECG: electrocardiography; ASA: American Society of Anesthesiologists.

Intraoperative data are presented in table 2 . No significant differences were observed between groups in intraoperative management, including total crystalloid volume administered. There were no significant differences in pRBC transfusion (6 *versus* 4 patients, p=0.735) or the number of patients requiring norepinephrine (18 *versus* 14 patients, p=0.457). Diuresis was similar in the Plasma-Lyte 148 and 0.9% saline groups (900 mL [380/1,750] *versus* 925mL [457/1,450], p=0.846).

**Table 2:** Intraoperative data

Variable	0.9% Saline Group (n=35)	Plasma-Lyte Group (n=33)	Difference between groups (95% CI)	p value
Surgery duration (min)^*^	360 (220/440)	270 (227/330)	+75 (0.0 to +135.0)	0.058
Anesthesia duration (min)^*^	480 (360/570)	370 (300/465)	+70 (0.0 to 140.0)	0.053
Crystalloid volume (mL)^*^	3000 (2000/4000)	2227 (1416/3000)	+515 (-100.0 to +1160.0)	0.107
Urine output (mL)^*^	900 (380/1750)	925 (457/1450)	+50 (-325.0 to +400.0)	0.846
Fluid balance (mL)^*^	+1785 (850/2570)	+1200 (634/1950)	+400 (-250.0 to +999.0)	0.216
Transfusion of red blood cells^†^ - %	6 (17.1)	4 (12.1)	+5 (-14.7 to +24.0)	0.735
Plasma transfusion^†^	0	1 (3)	-3 (-9.6 to +17.5)	0.485
Ephedrine dose (mg)^*^	0.0 (0.0/0.0)	0.0 (0.0/10.0)	0.0 (0.0 to 0.0)	0.052
Metaraminol dose (mg)^*^	1.0 (0.0/2.3)	0.0 (0.0/1.0)	0.0 (0.0 to 1.0)	0.075
Use of norepinephrine^†^ - %	18 (5.4)	14 (42.4)	+10 (-15.9 to +32.5)	0.457

^*^ Values are expressed as the median with the first and third quartiles and were analyzed using the Mann-Whitney U test; ^†^ Values are expressed as absolute and relative frequencies and were analyzed using the Chi-square and Fisher exact tests.

^‡^ Values are expressed as the median with the first and third quartiles and were analyzed using the Mann-Whitney U test and Hodges-Lehmann estimate.

95% CI: 95% confidence interval (Hodges-Lehmann estimate).

At the end of surgery, the Plasma-Lyte group had significantly higher arterial pH than the saline group (7.39±0.04 *versus* 7.35±0.05, p<0.001). Secondary outcomes showed that the Plasma-Lyte group had higher bicarbonate levels (22.5±1.8 *versus* 20.6±2.2mmol/L, p<0.001), less negative base excess (-1.6±2.3 *versus* -3.9±2.6, p<0.001), and lower chloride levels (109.1±6.6 *versus* 113.9±4.5 mmol/L, p=0.001).

The saline group had higher glucose levels (133 *versus* 119mg/dL, p=0.030) and slightly higher calcium levels (1.17±0.05 *versus* 1.14±0.04mmol/L, p=0.040). Sodium and potassium levels were similar between groups ( Table 3 ).

**Table 3:** Primary and secondary outcomes

Variable	0.9% Saline Group (n=35)	Plasma-Lyte Group (n=33)	Difference between groups (95% CI)	P value
Primary outcome				
pH^*^	7.35±0.05	7.39±0.04	-0.04 (from -0.06 to -0.02)	<0.001
Secondary outcomes				
HCO_3_^-^ (mmol/L)^*^	20.6±2.2	22.5±1.8	-1.83 (from -2.82 to -0.83)	<0.001
Base Excess^*^	-3.9±2.6	-1.6±2.3	-2.36 (from -3.57 to -1.16)	<0.001
Sodium (mmol/L)^*^	140.9±3.6	139.8±3.7	+1.03 (from -0.76 to +2.82)	0.255
Potassium (mmol/L)^*^	4.1±0.3	4.1±0.2	+0.05 (from -0.10 to +0.21)	0.480
Calcium (mmol/L)^*^	1.17±0.05	1.14±0.04	+0.02 (from +0.00 to +0.51)	0.040
Chlorine (mmol/L)^*^	113.9±4.5	109.1±6.6	+4.83 (from +2.09 to +7.58)	0.001
Lactate (mmol/L)^†^	1.4 (1.0/2.0)	1.3 (1.0/1.9)	0.9 (from -0.23 to +0.40)	0.595
Blood Glucose (mg/dL)^†^	133 (118.0/162.0)	119 (109.0/140.2)	+15.0 (from +1.00 to +28.0)	0.030

^*^ Values are expressed as mean and standard deviation and were analyzed using the Student’s t-test; ^†^ Values are expressed as median and quartiles and were analyzed using the Mann-Whitney U test and Hodges-Lehmann estimate of the median difference.

95% CI: 95% confidence interval.

We did not observe differences between groups regarding sodium, potassium, and lactate values.

There were no differences between groups regarding extubation performed in the operating room, length of hospital stay, length of ICU stay, new neurological deficit, or mortality (p>0.05) (Table 2S, Supplementary Material ).

No patient in either group received mannitol or hypertonic saline.

## DISCUSSION

This double-blind randomized controlled study demonstrated that Plasma-Lyte 148 provided significant metabolic advantages over 0.9% saline in patients undergoing elective neurosurgery, consistent with patterns reported in the international literature. With Plasma-Lyte, we observed superior arterial pH (7.39±0.04 *versus* 7.35±0.05, p<0.001), higher bicarbonate levels (22.5±1.8 *versus* 20.6±2.2mmol/L, p<0.001), less negative base excess (-1.6±2.3 *versus* -3.9±2.6, p<0.001), and lower chloride concentrations (109.1±6.6 *versus* 113.9±4.5mmol/L, p<0.001). These findings are virtually identical to those reported by Shrivastava et al., with similar methodology,^( 20 )^ as well as Dey et al. in elective craniotomies,^( 21 )^ and Sundaram et al. in aneurysm clipping,^( 69 )^ establishing a robust pattern of specific evidence for neurosurgery.

The remarkable consistency of these metabolic findings across multiple neurosurgical studies reflects a universal pattern observed in more than 50,000 patients in the general literature. The meta-analysis by Huang et al., involving 871 patients undergoing non-renal surgery, demonstrated similar differences: lower postoperative pH (mean difference 0.05, p<0.001) and lower base excess (mean difference 2.04, p<0.001) with saline.^( 65 )^ This pattern has been confirmed in diverse contexts, from the landmark study by Yunos et al. in critically ill patients^( 8 )^ to studies on renal transplantation^( 70 )^ and abdominal surgery,^( 71 )^ demonstrating that the metabolic advantages of balanced crystalloids extend across specific populations and clinical contexts.

In our study, no patient required mannitol or hypertonic saline. Administration of these agents to relieve intracranial pressure and cerebral edema could have resulted in hemodynamic and electrolyte changes that could not be attributed solely to the randomized solution.^( 72 )^

Our findings should be interpreted within the context of the evolving evidence regarding crystalloids. Although previous studies, such as SMART and SALT-ED, demonstrated not only metabolic advantages but also significant renal protection in more than 29,000 patients,^( 9 , 10 )^ more recent studies, such as BaSICS (2021) and PLUS (2022), revealed substantial equipoise, particularly in general critical populations.^( 11 , 13 )^ The PLUS study, which included 5,037 patients, demonstrated that despite expected metabolic advantages, there were no significant differences in mortality (21.8% *versus* 22.0%) or renal function between Plasma-Lyte 148 and saline.^( 13 )^ This evolution in evidence emphasizes the importance of contextualization and individualization of fluid therapy.

Elective neurosurgery represents a clinical context that is distinct from that of the general critical population studied in large clinical trials. Unlike traumatic brain injury, for which recent meta-analyses have raised concerns about balanced crystalloids,^( 12 )^ elective neurosurgery occurs in stable patients with intact blood-brain barrier and without the confounding factors typical of trauma, such as primary brain injury, edema, and hemodynamic instability.^( 16 , 17 )^ Our findings, which demonstrate metabolic advantages without compromising safety, align with this pathophysiological distinction and suggest that the benefits of balanced crystalloids can be obtained with equivalent safety in an elective neurosurgical setting.

The observed metabolic advantages have a solid physiological basis and potentially important clinical implications. Hyperchloremic acidosis induced by saline through a reduction in the strong ion difference, according to Stewart’s approach,^( 23 , 24 )^ can affect multiple organ systems. Sen et al. demonstrated that each 100 mEq increase in chloride load was associated with a 5.5% increase in death risk at 1 year, even after controlling for total fluid volume.^( 73 )^ In neurosurgery, where cerebrovascular acid-base regulation is critical for maintaining adequate cerebral perfusion,^( 74 , 75 )^ preservation of physiological acid-base balance may have neuroprotective implications, as suggested by recent experimental studies.^( 26 )^

In our study, serum ionic calcium levels were lower in the Plasma-Lyte 148 group than in the 0.9% saline group, although mean values in both groups were within the normal range. This may have occurred because of calcium chelation by plasma gluconate accumulation and the absence of calcium in Plasma-Lyte 148, similar to findings from previous studies,^( 19 , 21 , 76 )^ suggesting that serum calcium should be monitored in patients receiving Plasma-Lyte 148.

We found no significant differences in plasma concentrations of lactate, sodium, or potassium in both groups, consistent with findings from previous studies.^( 7 , 77 , 78 )^ Plasma lactate has been used as a marker of tissue perfusion in critically ill patients.^( 79 )^ The absence of differences in lactate levels between groups in our study indicated that the fluid management was equivalent and adequate to maintain optimum tissue perfusion, independent of the solution infused. Moreover, despite the different sodium concentrations between the two fluids studied (Plasma-Lyte 148, 140mEq/L; 0.9% saline, 154mEq/L), the sodium levels at the end of the procedure did not differ between groups, consistent with findings from other studies using similar methodology.^( 21 )^

O’Malley et al.^( 80 )^ compared Ringer’s lactate and 0.9% saline during kidney transplantation and found higher potassium levels in the 0.9% saline group (19% *versus* 0%), possibly as a result of hyperchloremic metabolic acidosis. Our study excluded patients with chronic kidney injury, pre-existing abnormalities of acid-base or electrolyte balance, or diuretic use, which are commonly encountered in renal transplant surgery and increase susceptibility to acidosis and hyperkalemia. This may explain why no difference in potassium levels was observed between groups.

We observed an increase in diuresis in the Plasma-Lyte 148 group, possibly due to the diuretic effect of gluconate, although this was not statistically significant. Similar findings have been reported by Roquilly et al.,^( 81 )^ who compared 0.9% saline with balanced solutions in patients with traumatic brain injury, and Chaussard et al.^( 76 )^ who compared Ringer’s Lactate with Plasma-Lyte 148 in patients with burns in intensive care.

Our findings contribute to the current refinement of fluid therapy, which has evolved from a “one-size-fits-all” approach to individualized strategies based on specific clinical contexts. Recent international consensus statements and guidelines, including European Society of Intensive Care Medicine (ESICM) 2024^( 5 )^ and PeriOperative Quality Initiative (POQI) 2024,^( 3 )^ emphasize the importance of this individualization. In elective neurosurgery, where controlled conditions fundamentally differ from general critical contexts, our findings suggest that the metabolic advantages of balanced crystalloids can be achieved safely, offering an optimized therapeutic strategy for this specific population.

Despite the exploratory nature of secondary outcomes, we observed no differences between groups in hospital or ICU length of stay, new neurological deficit, or mortality. Prior neurosurgical studies using a methodology similar to ours did not evaluate these parameters.^( 21 , 78 )^ We acknowledge that our sample size was calculated focusing solely on the primary outcome. Nevertheless, our study makes a novel contribution by evaluating these outcomes, serving as a foundation for future studies.

Although a significant difference in blood glucose levels was observed between groups, values remained within the normal range and did not require clinical intervention. Additionally, multiple univariate analyses increase the risk of family-wise error rate. Therefore, this difference may be random without clinical relevance. In a similar study, Abhiruchi et al. did not find differences in glucose levels between groups.^( 78 )^

This study has several strengths. As a double-blind, randomized controlled trial, it demonstrated high methodological quality, with low risk of bias across all evaluated domains. A comprehensive assessment using both the modified Jadad scale and the National Heart, Lung, and Blood Institute (NHLBI) Quality Assessment Tool confirmed the internal validity and reliability of our findings. The maximum scores achieved in both assessment tools indicate that our study meets the highest standards for clinical trial methodology and provides robust evidence for clinical decision-making.^( 82 , 83 )^

Nevertheless, certain limitations should be acknowledged. Although the sample size was sufficient to detect metabolic differences, it may be insufficient to evaluate rare clinical outcomes. The study population, restricted to elective neurosurgery, limits the generalizability of the findings to other neurosurgical contexts. Future studies with larger sample sizes and long-term follow-ups are needed to assess whether the observed metabolic advantages translate into sustained clinical benefits. Additionally, evaluation of specific biomarkers of brain injury and renal function could provide valuable mechanistic insights into the effects of different crystalloids in the neurosurgical context.

In conclusion, this study demonstrated that Plasma-Lyte 148 provides substantial metabolic advantages over 0.9% saline in elective neurosurgery, confirming the universal pattern observed in the international literature. These findings, obtained in a controlled context that fundamentally differs from the general critical population, contribute to the growing body of evidence supporting individualization of fluid therapy based on clinical context. In elective neurosurgery, where metabolic advantages of balanced crystalloids can be obtained with an equivalent safety profile, our findings offer valuable evidence for informed and personalized clinical decision-making. Future studies should focus on large, multicenter trials powered to detect clinical outcomes, investigation of long-term neurological effects, and exploration of optimal fluid management strategies across different neurosurgical populations. Additionally, the integration of goal-directed fluid therapy protocols with balanced crystalloids presents a promising avenue for optimizing perioperative care in patients undergoing neurosurgery.^( 51 , 84 )^ Our study provides the foundational evidence necessary to inform future investigations and contributes essential data to guide evidence-based crystalloid selection in contemporary neurosurgical practice.

## CONCLUSION

This randomized controlled trial demonstrated that Plasma-Lyte 148 provides superior acid-base homeostasis compared to 0.9% saline in elective neurosurgery, with statistically significant improvements in pH, bicarbonate, base excess, and chloride levels, while maintaining an equivalent safety profile. These findings support the use of balanced crystalloids in elective neurosurgical patients—a clinical context distinct from traumatic brain injury, where balanced solutions have shown concerning outcomes—and contribute essential evidence to support individualized perioperative fluid management.

## SUPPLEMENTARY MATERIAL

Figure 1SFlowchart of intraoperative fluid administration
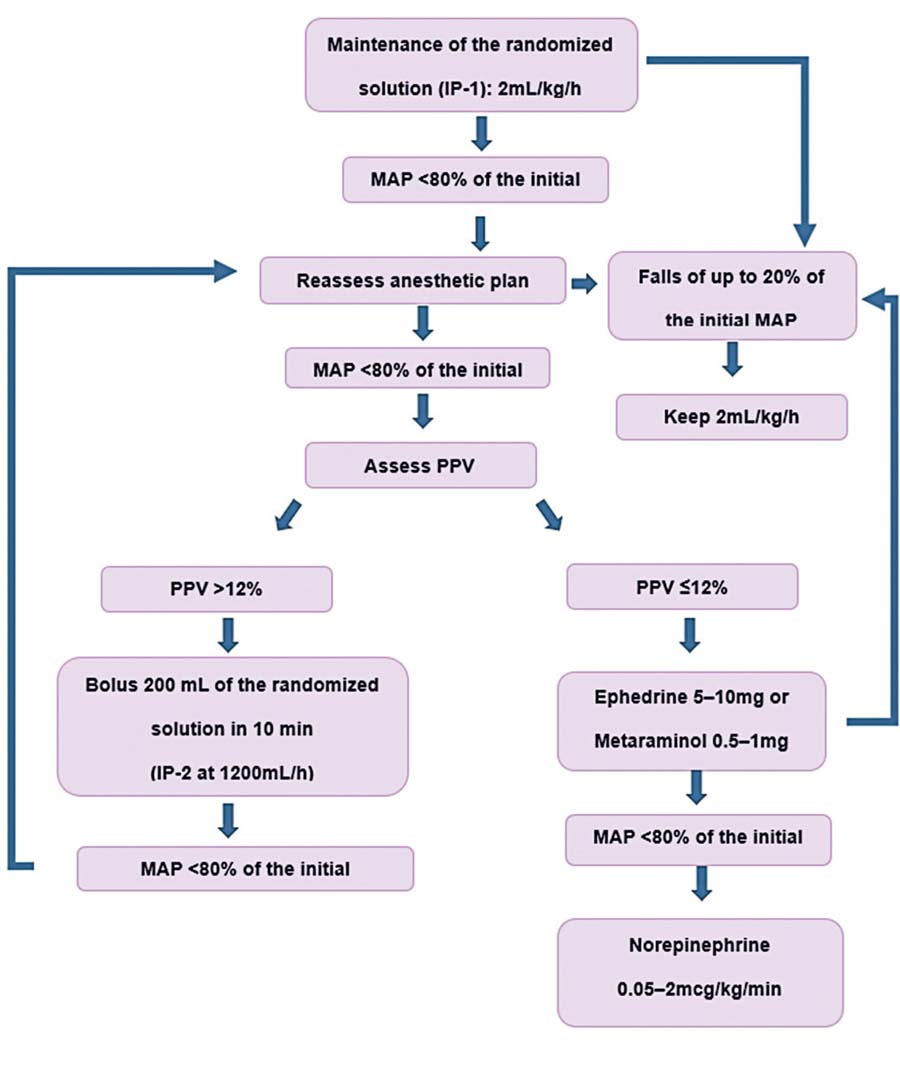
MAP: mean arterial pressure; Hb: hemoglobin; IP: infusion pump; PPV: pulse pressure variation.

Table 1S Reference values used for laboratory parameter assessment

**Table:** 

Reference values	
pH	7.35 to 7.45 ^(^ ^1^ ^,^ ^6^ ^)^
PaO2	90 to 100 mmHg ^(^ ^1^ ^,^ ^6^ ^)^
PaCO2	30 to 35 mmHg ^(^ ^1^ ^,^ ^6^ ^)^
HCO3	22 to 26 mmol/L ^(^ ^1^ ^,^ ^6^ ^)^
Base excess	-3.5 to +3.5 ^(^ ^1^ ^,^ ^6^ ^)^
Sodium	135 to 142 mmol/L ^(^ ^1^ ^,^ ^2^ ^,^ ^7^ ^)^
Potassium	3.5 to 5.3 mmol/L ^(^ ^1^ ^,^ ^2^ ^,^ ^8^ ^)^
Ionic calcium	1.11 to 1.4 mmol/L ^(^ ^1^ ^,^ ^3^ ^,^ ^9^ ^)^
Chlorine	97 to 107 mmol/L ^(^ ^2^ ^,^ ^10^ ^)^
Lactate	0.5 to 1.8 mmol/L ^(^ ^4^ ^,^ ^5^ ^,^ ^11^ ^)^
Glycemia	100 to 180 mg/d ^(^ ^7^ ^,^ ^12^ ^,^ ^13^ ^)^

Table 2S Interventions and events in the 7 postoperative days

**Table:** 

Variable	0.9% Saline Group	Plasma-Lyte Group	Difference between groups (95% CI)	p value
Extubation in-room ^*^ - %	29 (82.9)	31 (93.9)	-11.0 (from -7.4 to +28.9)	0.260
Hospital time (days) ^†^	4.0 (3.0/9.0)	4.0 (3.0/6.0)	0.0 (from -1.0 to +1.0)	0.642
ICU time (days) ^†^	0.0 (0.0/2.0)	0.0 (0.0/0.0)	0.0 (from 0.0 to 0.0)	0.065
New neurological deficit ^*^ - %	5 (14.3)	6 (18.2)	-3.9 (from +15.9 to +23.8)	0.749
Death ^*^ - %	4 (11.4)	1 (3.0)	-8.4 (from -8.0 to +24)	0.357

^*^ Values are expressed as absolute and relative frequencies and were analyzed using the Chi-square and Fisher exact tests; ^†^ Values are expressed as the median with the first and third quartiles and were analyzed using the Mann-Whitney U test and Hodges-Lehmann estimate.

95%CI: 95% confidence interval; ICU: intensive care unit.
